# The Use of Esterified Hyaluronic Acid Matrix in the Setting of a Large Marjolin Ulcer Excision and Reconstruction: A Case Report

**DOI:** 10.7759/cureus.77454

**Published:** 2025-01-15

**Authors:** Kimiya Taji, Vaibhav Varma, Daniel Walk, Shay B Dean

**Affiliations:** 1 Surgery, St. George's University School of Medicine, St. George, GRD; 2 General Surgery, HCA Houston Healthcare Kingwood/University of Houston, Kingwood, USA; 3 Plastic Surgery, Southern California University of Health Sciences, Los Angeles, USA; 4 Plastic and Reconstructive Surgery, Dean Plastic Surgery Associates Inc., Los Angeles, USA

**Keywords:** chronic burn scar, dermal substitute, esterified hyaluronic acid matrix, hyaluronic acid, marjolin ulcer, plastic and reconstructive surgery, split-thickness skin graft (stsg), squamous cell carcinoma

## Abstract

Marjolin ulcers (MUs) are a rare complication of chronically inflamed wounds and scars, with the most common histology being squamous cell carcinoma. MUs have high rates of metastasis and recurrence and a poor prognosis. Due to their aggressive nature, such cutaneous growths require radical excision and subsequent grafting. In large MUs, the use of a multi-stage approach to reconstruction provides better wound healing and graft recipient sites. An additional modality to further the results of the reconstructive approach includes the use of an esterified hyaluronic acid matrix (eHAM) prior to the placement of split-thickness skin grafts. eHAM, frequently used in wounds and deep burns, provides a scaffold for granulation tissue formation thus furthering the strength of graft recipient sites. We present a case of a large MU at the site of a long-standing burn scar that was excised with clear margins and reconstructed with stage skin grafting using eHAM.

## Introduction

Marjolin ulcers (MUs), which typically present as fast-growing squamous cell carcinoma (SCC), are a rare and aggressive complication of chronic inflammatory processes. MUs constitute approximately 2% of SCC and disproportionately affect males more than females, occurring at a ratio of 2:1 [1]. The etiology of MU is considered to be the re-epithelization of scars due to continuous inflammation and decreased blood flow. Subsequent loss of immune integrity of the wound site contributes to the aggressive growth of MUs [1]. Typical warning signs of MU include progression of previous non-healing wounds to an ulcerative appearance with rolled borders, itching, burning, and foul-smelling pus secreted. If MU is suspected, timely biopsy is essential and, if confirmed, excision with clear margins is necessary. The reconstructive approach for the excision site depends on the size and location of the defect. Smaller ulcers may be removed using wide 1-2 cm excision [2]. Large MUs may require wide local excision in combination with staged split-thickness skin grafting (STSG) as one option for reconstruction.

Esterified hyaluronic acid matrix (eHAM) is often utilized in patients with chronic ulcers, scars, and traumatic wound defects. Hyaluronic acid (HA), a polysaccharide, plays a large role in wound integrity and the extracellular matrix in various tissues such as the skin, eyes, and joints. More specifically, HA provides a scaffold for native cell migration and an increase in pro-inflammatory cytokines providing fibroblast proliferation. Clinical advantages of using eHAM include promoting wound healing with secondary intention and enhanced support of angiogenesis. One disadvantage to STSG includes the requirement of a robust blood supply at the recipient site [3]. Often, patients suffering from MUs have a concomitant contracture of surrounding skin and poor blood supply, making STSG susceptible to complications and failure. The use of eHAM in this context provides a more viable graft recipient wound bed to promote healing and better outcomes.

This article was previously presented as a poster at the 2024 NMA Annual Scientific Convention and Assembly on August 3-7, 2024.

## Case presentation

A 68-year-old male presented to our practice for surgical consultation after discovering a large MU on his left posterior thorax. In childhood, the patient sustained full-thickness burns to his posterior shoulder and entire posterior thorax. At the time, the patient underwent skin grafting to the entirety of the burn area resulting in significant scarring. Years before the office visit, a scaly, non-healing mass/ulceration developed with radial expansion progressively worsening over time. The physician at the time informed the patient that the ulceration was positive for SCC. The patient underwent further testing with a positron emission tomography (PET) scan testing for distant metastasis. The PET scan was negative, and the patient was referred for mass excision and reconstruction. The patient had no significant prior medical history and denied a history of smoking.

**Figure 1:**
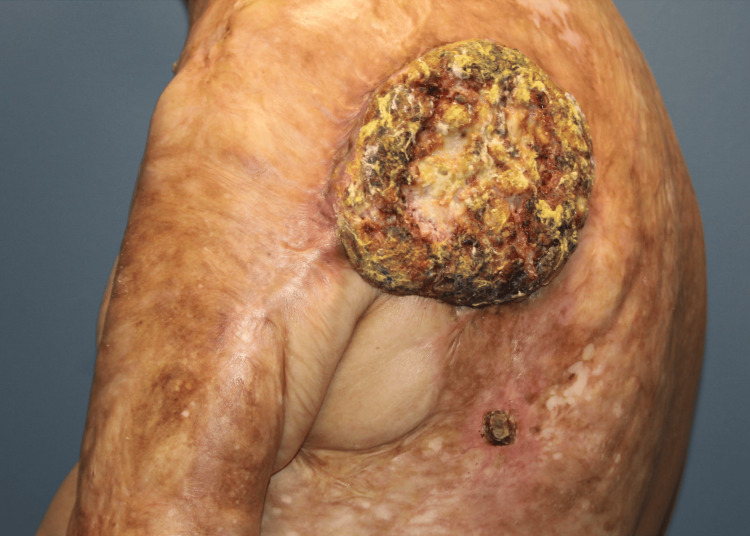
Marjolin ulcer

At his first visit, the patient complained of a strong odor and discomfort from the large mass (Figure 1). Upon initial examination, a 40 cm x 30 cm large fungating SCC with central necrosis was apparent. The wound exhibited large necrotizing features with small satellite lesions surrounding the mass. Based on the size and location of the mass, the necessity of a staged reconstruction was explained to the patient, with the first stage being mass excision and placement of an eHAM, followed by subsequent debridement with STSG for definitive closure.

**Figure 2:**
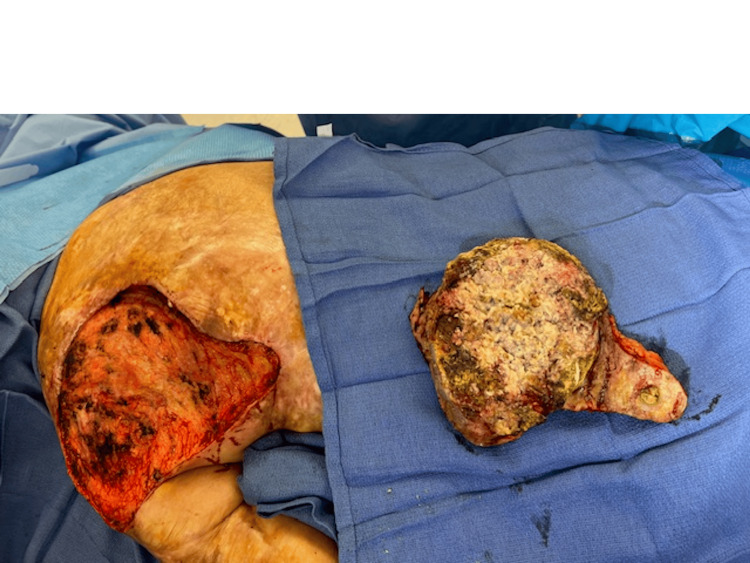
Resection of Marjolin ulcer

**Figure 3:**
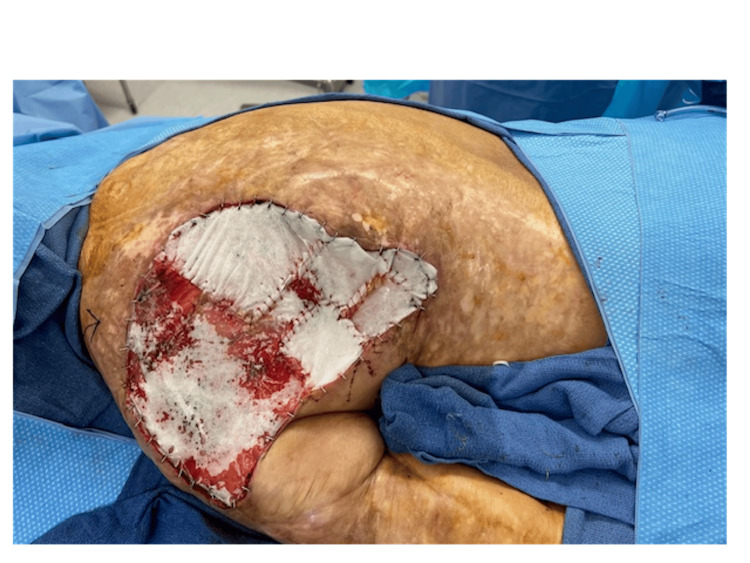
Placement of esterified hyaluronic acid matrix with fixation

The initial stage of the reconstructive process began with circumscribing the MU down to the latissimus dorsi muscle fascia. A fascial plane was utilized, and meticulous dissection of all dimensions of the ulceration including satellite lesions was performed (Figure 2). Subsequently, margins measuring 2 cm x 2 cm were excised, including medial, lateral, superior, and inferior aspects, and sent to pathology. The area was then irrigated, and careful placement of eHAM was completed with fixation using a 3-0 chromic suture (Figure 3). The wound was subsequently covered with bacitracin ointment, Xeroform gauze, and absorbable nonadherent dressing pads. The patient's home health nurse was advised to perform dressing changes until the second procedure was completed. The patient experienced no complications in the first stage of the reconstructive process.

**Figure 4:**
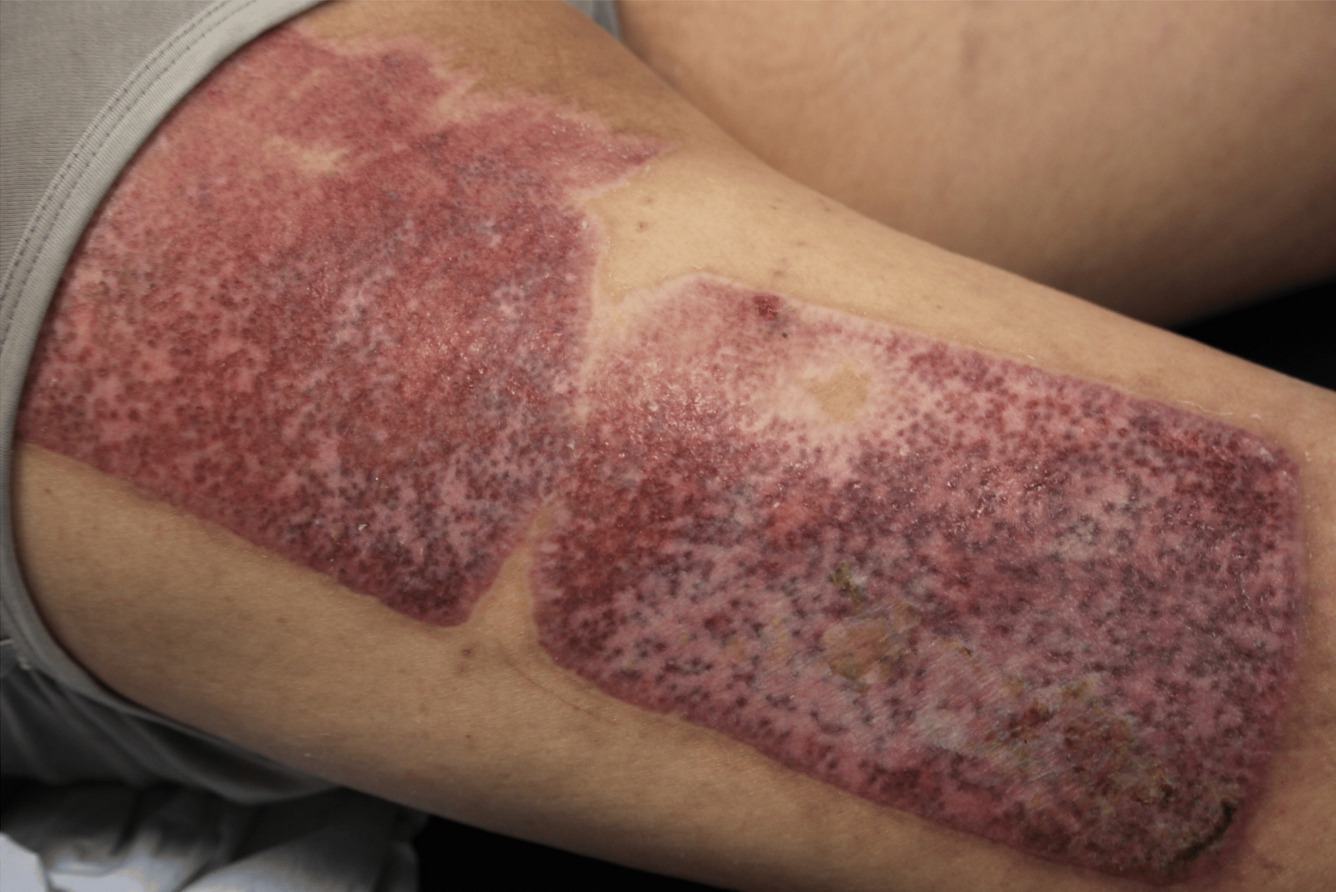
Healed split-thickness skin graft donor site four weeks postoperatively

Three weeks after mass excision, the patient returned for debridement and STSG. eHAM placement showed adequate wound healing and granulation tissue, indicating a favorable graft recipient site. The patient was placed under general anesthesia and debridement of the open wound was initiated. The patient’s left posterior shoulder and thorax displayed satisfactory punctate bleeding and granulation tissue throughout. A STSG was harvested from the left posterior thigh and buttocks (Figure 4). The harvested skin graft was then placed on the defect and secured using staples. Epinephrine-soaked laps were placed on the site to prevent further bleeding and promote hemostasis. A wound vacuum-assisted closure set to 125 mmHg suction was placed as a bolster. The donor graft site was then dressed with bacitracin ointment, Xeroform gauze, absorbable nonadherent dressing pads, and paper tape.

**Figure 5:**
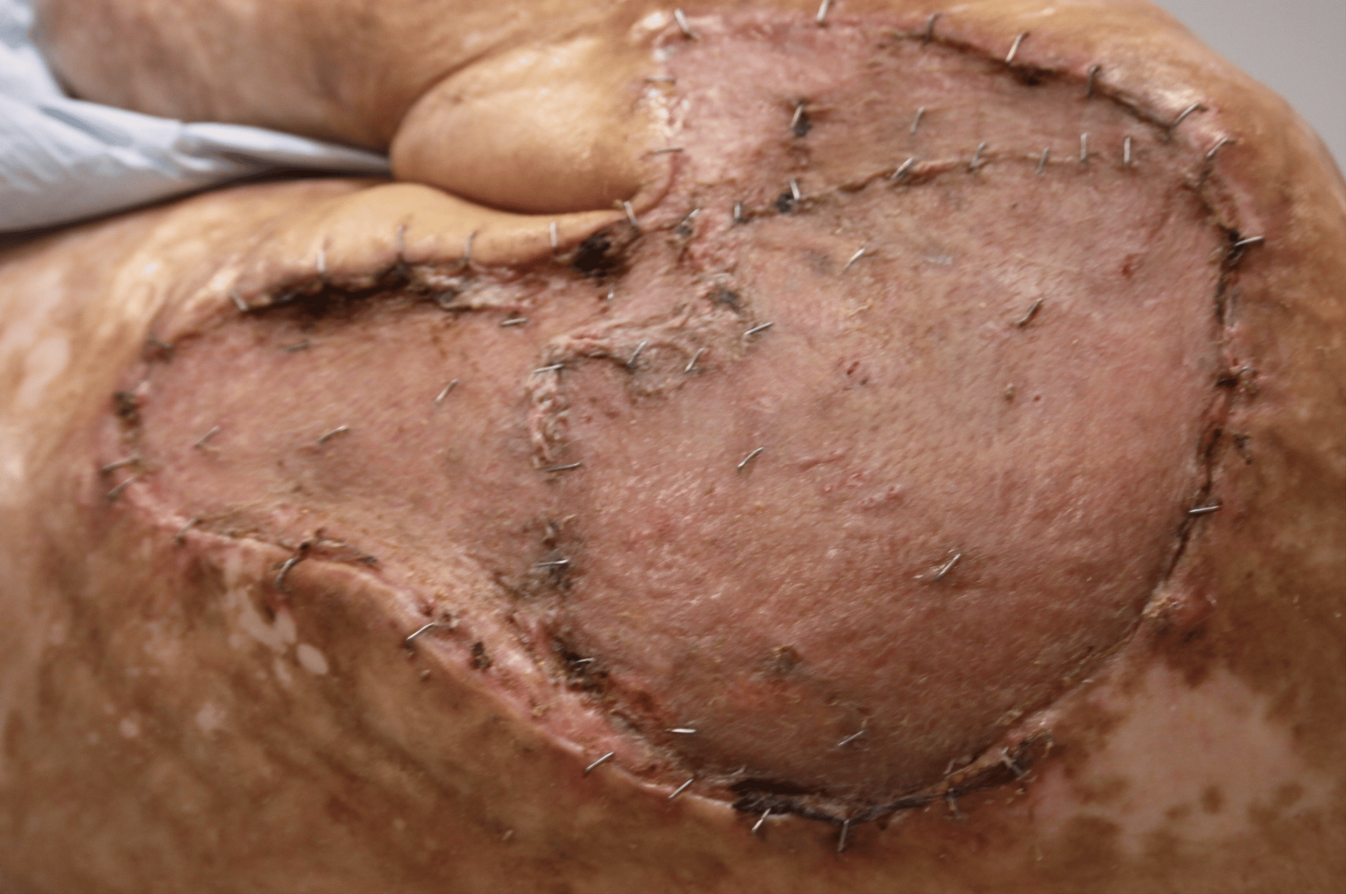
Recipient graft site two weeks postoperatively

**Figure 6:**
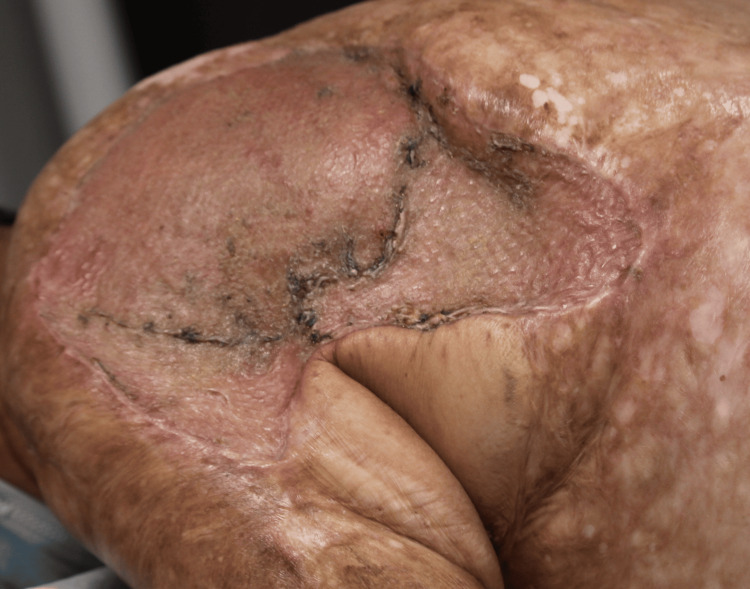
Adequate epithelialization of recipient graft site

The patient’s postoperative course for both procedures showed no signs of complications such as infection, dehiscence, or necrosis. During follow-up, the patient’s right shoulder recipient graft site showed well-approximated edges and signs of adequate epithelialization (Figures 5, 6). Pathology report following excision showed well-differentiated SCC with minimal atypia and negative margins. On physical examination, the patient exhibited a small amount of diminished abduction postoperatively (15 degrees) due to scar contracture in the axilla. Plan to perform Z-plasty to release the scar contracture was proposed to the patient; however, the patient was lost to follow-up.

## Discussion

MU, characterized by malignant transformation in chronic wounds or burn scars, present a unique set of challenges due to their aggressive nature and the complex tissue environment in which they develop. Thus, the utilization of eHAM in the surgical excision and staged reconstruction of an MU at the site of a burn scar represents a significant reconstructive surgery procedure.

eHAM is a modified form of HA, where the carboxyl groups are esterified to enhance its stability and prolong its presence in the tissue [4]. The esterification process enhances the molecule's stability and persistence within the wound site [5], thus ensuring long-term support for the regenerating tissue. The structure of eHAM allows it to draw water into the wound bed, maintaining a moist environment that is crucial for effective healing. Thus, the matrix is designed to support tissue regeneration and wound healing by creating a conducive environment for cellular activities, such as capillary ingrowth and cellular invasion, by maintaining tissue hydration and facilitating cell migration [6].

eHAM is widely used in treating various types of wounds, including burns and chronic wounds [7]. Its ability to promote tissue regeneration and reduce the time to wound closure makes it an effective application in burn care. It is particularly useful in preparing burn wounds for subsequent skin grafting procedures [8]. In plastic and reconstructive surgery, eHAM is utilized to enhance the healing of surgical wounds, especially where there is significant tissue loss or damage and tissue regeneration is a priority. Its application can help in the regeneration of tissues in areas that have been surgically debrided or exposed due to trauma or burns [9]. In creating an effective healing environment, eHAM can help reduce scar formation and improve both functional and aesthetic outcomes [10]. Overall, the application of eHAM has been shown to provide notable benefits such as accelerating the healing process in burns, epithelial surgical wounds, and chronic wounds [11].

Our findings demonstrated that the use of an eHAM facilitated effective wound healing and tissue regeneration post-excision. The matrix acted as a biocompatible scaffold that supported cellular infiltration and neovascularization. This contributed to a more robust and rapid healing process, as evidenced by the patient’s postoperative recovery. Additionally, the anti-inflammatory properties of HA may have played a crucial role in creating a favorable healing environment. This likely helped in reducing local inflammation and the risk of infection, which are critical factors in the management of surgically treated MU. Fundamentally, the use of eHAM in this case appeared to improve the initial post-procedure course and provided a strong foundation for later skin grafting. The patient experienced minimal complications and exhibited satisfactory functional and aesthetic outcomes.

While the results from this case are promising and suggest further consideration and implementation of eHAM in plastic and reconstructive procedures, this is a single-case report, and any significant clinical benefits require validation through larger, controlled studies. Further research should focus on comparing the outcomes of other reconstructive methods, especially using alternative dermal substitutes, with those incorporating esterified HA matrices to establish definitive benefits and potential limitations. Moreover, long-term follow-up is necessary to assess the durability of the reconstructed tissue and monitor for any signs of recurrence. The cost-effectiveness and accessibility of eHAM should also be considered in broader clinical practice.

## Conclusions

MUs present a significant challenge in terms of treatment and reconstruction. Often occurring at burn scars and requiring wide excision, surgical wounds resulting from MU removal can be difficult to close primarily or with immediate skin grafting. The incorporation of an eHAM in the surgical management of MUs at burn scar sites appears to enhance wound healing and tissue reconstruction. Furthermore, this allows for a staged reconstruction with definitive skin grafting after a robust wound bed is available. This approach holds promise for improving patient outcomes in complex reconstructive surgeries, warranting further investigation and application in clinical settings. It is possible that a variety of reconstructive surgeries may benefit from a staged approach with eHAM as an intermediate step.
